# Tissue Engineering and Photodynamic Therapy: A New Frontier of Science for Clinical Application -An Up-To-Date Review

**DOI:** 10.3389/fbioe.2022.837693

**Published:** 2022-06-15

**Authors:** Mariza Aires-Fernandes, Camila Fernanda Amantino, Stéphanie Rochetti do Amaral, Fernando Lucas Primo

**Affiliations:** Department of Bioprocess and Biotechnology Engineering, School of Pharmaceutical Sciences, São Paulo State University–UNESP, Araraquara, Brazil

**Keywords:** tissue engineering, bioprinting, skin model, photodynamic therapy, photobiostimulation

## Abstract

Tissue engineering (TE) connects principles of life sciences and engineering to develop biomaterials as alternatives to biological systems and substitutes that can improve and restore tissue function. The principle of TE is the incorporation of cells through a 3D matrix support (scaffold) or using scaffold-free organoid cultures to reproduce the 3D structure. In addition, 3D models developed can be used for different purposes, from studies mimicking healthy tissues and organs as well as to simulate and study different pathologies. Photodynamic therapy (PDT) is a non-invasive therapeutic modality when compared to conventional therapies. Therefore, PDT has great acceptance among patients and proves to be quite efficient due to its selectivity, versatility and therapeutic simplicity. The PDT mechanism consists of the use of three components: a molecule with higher molar extinction coefficient at UV-visible spectra denominated photosensitizer (PS), a monochromatic light source (LASER or LED) and molecular oxygen present in the microenvironment. The association of these components leads to a series of photoreactions and production of ultra-reactive singlet oxygen and reactive oxygen species (ROS). These species in contact with the pathogenic cell, leads to its target death based on necrotic and apoptosis ways. The initial objective of PDT is the production of high concentrations of ROS in order to provoke cellular damage by necrosis or apoptosis. However, recent studies have shown that by decreasing the energy density and consequently reducing the production of ROS, it enabled a specific cell response to photostimulation, tissues and/or organs. Thus, in the present review we highlight the main 3D models involved in TE and PS most used in PDT, as well as the applications, future perspectives and limitations that accompany the techniques aimed at clinical use.

## Introduction

Tissue engineering (TE) is an interdisciplinary field, integrating engineering and medicine, that purpose to develop biological substitutes that will replace, repair or improve tissues and organs (Hasan et al., 2018). In 1970s was the first time that the concept of tissue engineering was introduced, by a pediatric orthopedic surgeon at Boston Children’s Hospital, W. T. Green, who performed several experiments aimed to generate cartilage from chondrocytes seeded in bone spicules (Melville et al., 2019). Twenty years later, TE was described by Robert Langer and Joseph Vacanti as “an interdisciplinary field that applies the principles of engineering and the life sciences toward the development of biological substitutes that restore, maintain, or improve tissue function” (Melville et al., 2019). In 2003, the Tissue Engineering Regenerative Medicine International Society was created, representing a huge milestone for the field, uniting scientists from all over the world to share and collaborate on their research, resulting in significant improvements (Melville et al., 2019).

**FIGURE 1:**
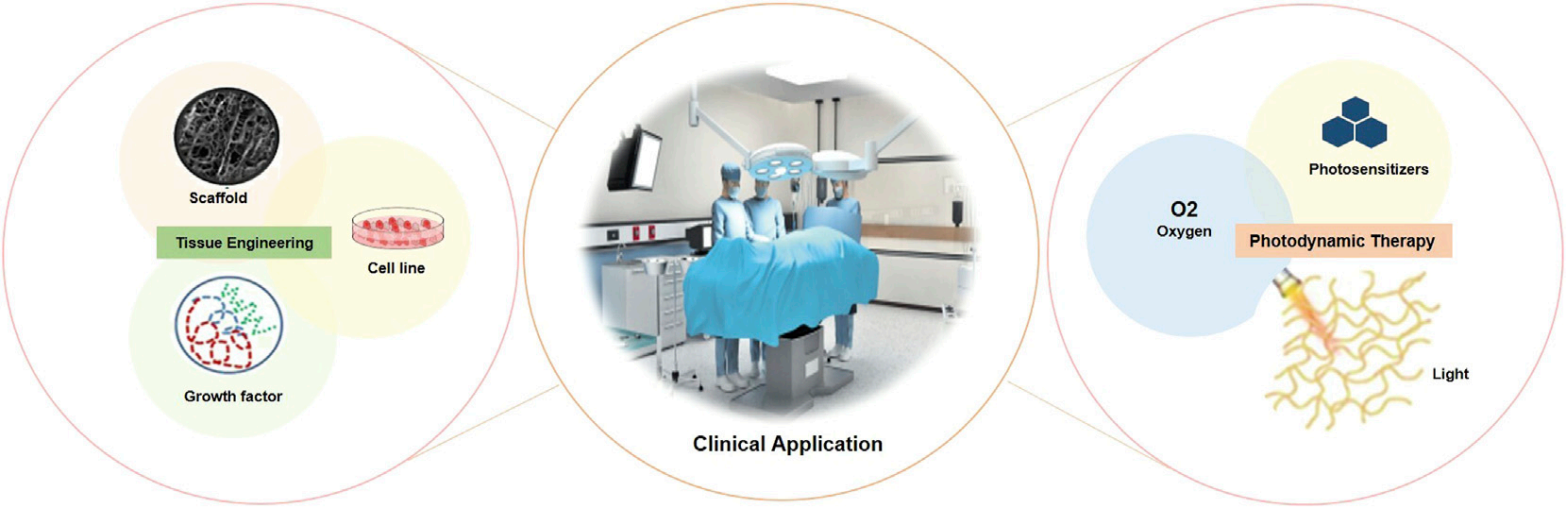
Main factors involved in the development of Tissue Engineering and PDT for clinical application. The cells that will be cultured are selected, along with the biomaterial to be used for the development of the scaffold, followed by the insertion of regulatory signals, such as growth factors. Photodynamic Therapy (PDT) is based on the combination of the photosensitizer (PS) through monochromatic light at a specific wavelength (λ) and molecular oxygen (O_2_) (Source: Authors own elaboration).

TE is a field that is growing rapidly, providing new tools to manage complex diseases, and is a promising alternative to tissue harvesting, artificial tissues, and prostheses, since there are still high levels of graft rejection, low availability or unavailability of organ donation (Bodnar et al., 2018; Blum et al., 2021). In Figure 1 is shown the three factors that are essential for successful tissue regeneration, a combination of scaffolds or a framework, cell signaling and cells (Melville et al., 2019). To create a microenvironment of the human body, an extracellular matrix (EMC) is required, forming the basis of all organs and tissues ((Maheshwari et al., 2019).

In this scenario, 3D cell culture creates a microenvironment that can simulate the EMC found in the human body, taking into account morphological, biochemical, and mechanical factors (Fitzgerald et al., 2015; Asadi et al., 2020).

3D cell culture method enables *in vivo* conditions can be mimicked (Shao et al., 2021). For this, 3D cell culture using scaffolds has been improved in order to simulate the complexity of tumors *in vivo* (Shao et al., 2021). Therefore, the 3D models developed provide soluble gradients and allow the distribution of adhesions in all three spatial dimensions without polarity (Herreros-Pomares et al., 2021; Shao et al., 2021).

There are several types of platforms for 3D culture such as cell biology-based models (spheroids and organoids) and engineering-based models (scaffold and microfluidic platforms), cell biology-based have the advantage of having a greater similarity in the early details of cell development *in vivo*, while engineering-based models have better organization and composition of materials to develop ideal conditions important in tissue reconstruction (Zhuang et al., 2018). The methods approached through TE are limited mainly by the lack of appropriate techniques to develop physiological architectures that can mimic the EMC, in addition to the lack of control of cellular functions and their numerous properties (Hasan et al., 2018).

Photodynamic Therapy (PDT) is a minimally invasive therapeutic modality used for the treatment of several diseases, including cancer and non-malignant lesions (Mohammad-Hadi et al., 2018). Raab and von Tappeiner first introduced the term “photodynamic effect” into the literature through studies that showed that certain classes of dyes can sensitize microorganisms when exposed to sunlight, leading to cell death (Kessel, 2019). The advances were even greater when a group of physicians from the Mayo Clinic demonstrated that by employing a hematoporphyrin derivative, the fluorescence in the tumor tissues tended to increase, and the acronym “HPD” was used to refer to the material (Kessel, 2019). After numerous advances in the field, the terminology “Photodynamic Therapy” was introduced, based on the words used by von Tappeiner (Kessel, 2019).

The mechanism used by PDT consists in the interaction between a photosensitizer (PS) and a specific wavelength (Figure 1) of light in the presence of oxygen. The interaction leads to the formation of reactive oxygen species (ROS) and free radicals, such as singlet oxygen (^1^O_2_), that lead to the destruction of the target cells or tissue (Qidwai et al., 2020; Sun et al., 2020). Generally, studies using PDT is performed in monolayer, in other words, two-dimensional (2D) *in vitro* models, with advantages related to simplicity of application and reliability (Wu et al., 2020a; Demir Duman et al., 2020). However, the use of these models ultimately misses the cellular interactions with the EMC and does not reproduce the microenvironment correctly (Demir Duman et al., 2020). The use of animal studies also has limitations, such as the cost and the time of the experiment, which are usually long. In order to circumvent these factors in both techniques, more and more investment is being made in 3D culture models, where the microenvironment is optimally reproduced (Demir Duman et al., 2020). A direct advantage related to the use of PDT and his dependence on oxygen, is that the use of 3D models can incorporate the hypoxia that occurs in several tissues, like cancerous tissue (Demir Duman et al., 2020).

The present article seeks to provide a broad review of Tissue Engineering and Photodynamic Therapy, highlighting the main 3D models involved in TE and the most commonly used photosensitizers in PDT, as well as the applications, future prospects, and limitations that accompany both techniques.

## Tissue Engineering

### Background

The advent of tissue engineering (TE) represents the intersection of a distinct areas, including clinical medicine, engineering and science, for the development of biological models that can be applied to improve, maintain or restore of tissue structures that were deteriorated or lost due to diseases such as cancer or trauma (Langer and Vacanti, 1993)**.**

In this context, one of the first publications found in PubMed referring to the term tissue engineering was described Bell and colleagues in 1981, they designed a tissue-engineered 3D human skin equivalent composed by dermal and epidermal layers (Bell et al., 1981). In 1984, the accidental development of an endothelium-equivalent membrane under the surface of a long-established synthetic ophthalmologic prosthesis was described (Wolter and Meyer, 1985).

After understanding the concept of tissue engineering, it is necessary to show how the 3D based models are composed. In this sense, the basic components of Tissue Engineering are: cell sources and management, development of scaffolds and substances that induce cell growth and differentiation (Guiro and Arinzeh, 2015; Hapach et al., 2019).

### Components of Tissue Engineering

The cell sources (not necessarily stem cells) used in TE that include autologous (differentiated cells), allogeneic (differentiated cells), adult stem cells/progenitors, embryonic stem cells (Al-Himdani et al., 2017).

There are several established techniques for developing TE-based 3d models to mimic current *in vivo* conditions. The models can be divided into cells cultivated as multicellular aggregates (spheroids) and cells incorporated in supports of natural or synthetic origin (scaffolds) (Guiro and Arinzeh, 2015; Naahidi et al., 2017; Brancato et al., 2020). The choice of scaffold must be carefully evaluated. The ideal scaffold should provide an architecture that allows for the attachment, migration, proliferation and differentiation of cells while enabling cell reorganization into a functional 3d network (Ceylan and Bölgen, 2016; Lanza et al., 2020).

Scaffolds of natural origin have the advantage of having better biocompatibility, less toxicity and can be prepared from natural polymeric materials, such as collagen, chitosan, glycosaminoglycans, fibroin, agarose, alginate and starch (Colley et al., 2011; Lv et al., 2017; Pal et al., 2019). While scaffolds of synthetic origin have greater versatility, ease of reproduction and therefore can be processed more easily than those of natural origin, and can be formed from polyglycolic acid, polylactic acid, polyorthoester and their copolymers or blends, as well as the aliphatic polyester polycaprolactone (Colley et al., 2011; Lv et al., 2017; Pal et al., 2019). There are also scaffolds based on ECM: allogeneic, xenogenic acellular dermis and others (Colley et al., 2011; Lv et al., 2017; Pal et al., 2019).

There are several advantages of scaffold-free 3D cell cultivation, such as the possibility of co-culture; low cost and high throughput screening approach. On the other hand, the absence of a scaffold makes it impossible for the cell-cell and cell-ECM interactions to be mimicked *in vitro*, as well as the control over the size of the spheroids/organoids obtained (Brancato et al., 2020). On the other hand, scaffolds can overcome some of these limitations, as it is possible to be cultivated in co-culture, there is a wide variety of materials, as well as a decellularized matrix, possibility of customization and the commercial availability of scaffolds. Despite all the advantages of scaffolds it must be considered that depending on the manufacturing technique the cost can be high, cell removal can be difficult in the case of scaffolds based on MEC, and the high-throughput screening options limited (Guiro and Arinzeh, 2015; Naahidi et al., 2017; Pal et al., 2019; Brancato et al., 2020).

### Approaches and Methods Available for Designing 3D Culture Models

The *in vitro* study models are mostly based on cell culture in two dimensions (2D), since the investigation in these models is more accessible and can be easily reproduced (Ceylan and Bölgen, 2016). However, 2D systems have several limitations, as they do not have the necessary complexity in their structural organization, in addition to the absence of connective tissue, essential factors to mimic the model/target organ (Song et al., 2014). In addition, 2D model studies often show false-positive responses, so it is necessary to use *in vivo* models to confirm the result. However, the use of animals has ethical dilemmas and costly procedures (Alemany-Ribes et al., 2013; Doke and Dhawale, 2015).

In this sense, the search for advanced models for alternative biological tests becomes indispensable. The developed models can be used for various purposes, from studies mimicking healthy tissues and organs as well as to simulate and study different pathologies (Guiro and Arinzeh, 2015).

In this sense, the search for advanced models for alternative biological tests becomes indispensable. The developed 3D models can be used for various purposes, such as studies mimicking healthy tissues and organs, simulation and study of different pathologies, as well as drug delivery assessment (Groeber et al., 2016; Magdeldin et al., 2017; Bourland et al., 2018; Nishiguchi et al., 2018; Grifno et al., 2019; Pal et al., 2019; Woappi et al., 2020).

## Photodynamic Therapy

**FIGURE 2:**
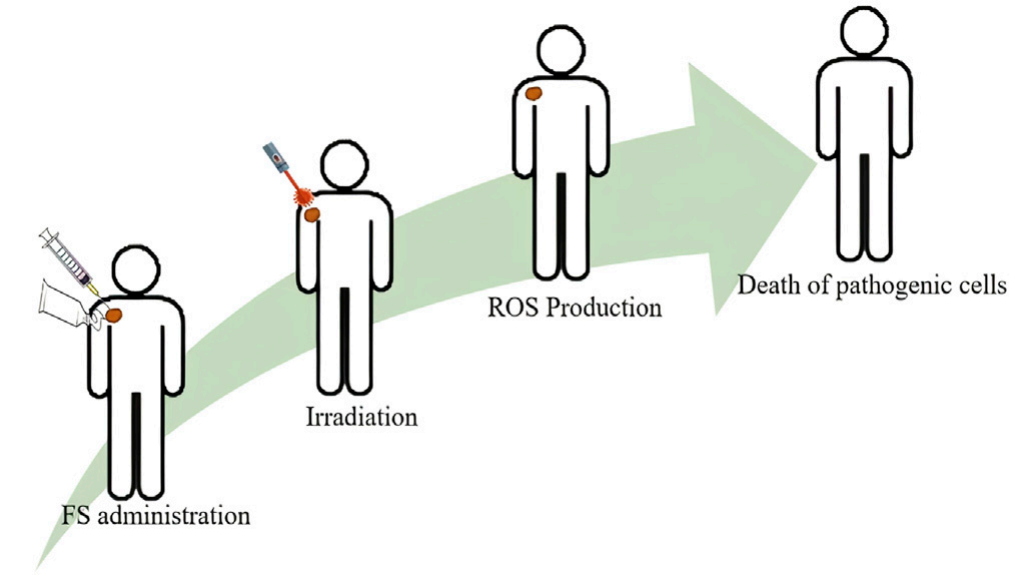
Basic Principle of Photodynamic Therapy (Source: Authors own elaboration).

Photodynamic therapy (PDT) is a therapeutic method that has been used in the treatment of several diseases, either as a single therapy or as a complement to conventional therapeutic protocols. This therapy is widely accepted by patients because it is less invasive than conventional ones, in addition to having few side effects and pain reduction. In addition, due to its therapeutic simplicity, it allows application in an outpatient setting, without the need for surgery (Li et al., 2017; Luo et al., 2017). The PDT mechanism is based on the correct combination of three components: photosensitizer (PS), monochromatic light at a specific wavelength (λ) and molecular oxygen (O_2_) dissolved in a biological medium, PS is administered systemically, topical or oral, followed by exposure to visible light, resulting in a series of reactions that result in the death of target pathogenic cells (Figure 2), the three components do not show toxicity when separated (Calixto et al., 2016). After the internalization of the PS in the cells, irradiation is performed with a laser or LED at the wavelength where PS has greater energy absorption, the PS absorbs this energy and is excited to the singlet and triplet excited states, followed by an energy transfer to O_2_, which leads to the production of reactive oxygen species defined as ROS that attack specific centers within cell systems, triggering the death of these tissues by processes of cell necrosis and/or apoptosis (Dai et al., 2012).

**FIGURE 3:**
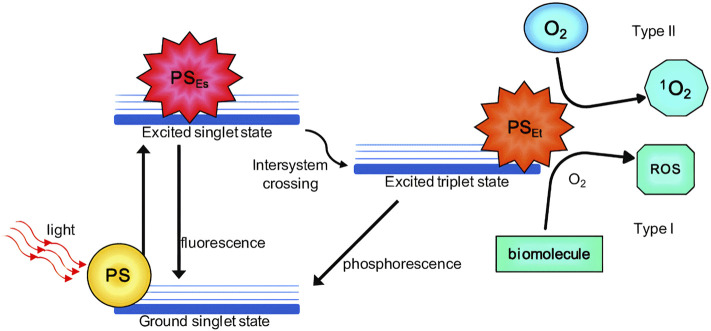
Type I and Type II reactions in PDT (photodynamic therapy). Schematic Jablonski’s diagram showing PDT’s mechanism of action. PS: photosensitizer; PSEs: PS excited singlet state; PS_Et_, PS excited triplet state; ROS, reactive oxygen species; ^1^O_2_, singlet oxygen. Source (Calixto et al., 2016).

After the absorption of a photon of light, PS leaves the lower energy ground state (S^0^) and passes to the higher energy singlet state (S^1^–S^n^), PS tends to return to the lower energy state, with this, part of the absorbed energy is used to return to the S0 state, through the physical relaxation process known as internal conversion or by radiative processes such as fluorescence emission (Figure 3) (Benov, 2015). However, part of the energy can be transferred by the Intersystem conversion mechanism, where the PS passes from the excited states S^1^/S^n^ and occupies the triplet excited state (T^1^) of lower energy which can also be returned directly to S0 by the internal conversion process or through the radiative process of emission of phosphorescence, or triggering a series of photochemical interactions that give rise to two known photodynamic mechanisms (Type I and II) (Foot, 1991; Kwiatkowski et al., 2018).

In the Type I mechanism, PS is in the T1 state, there is a transfer of energy to the biomolecules or the abstraction of a hydrogen atom can also occur, in both cases these reactions result in the formation of free radicals or radical ions, in turn, these reduced species can transfer electrons to molecular oxygen diffused in the medium (which is in its triplet state), which leads to the generation of reactive oxygen species (ROS), hydrogen peroxides, anion superoxides. These reactions are outlined below (Benov, 2015).

Type I mechanism–Redox reactions with biomolecules

P0→P1→P3

P3+S→P+·+S-·(energy transfer)

S-·+O23→S+O2-·→HO·+HO·

P = photosensitizer; S = organic substrate; + = cation and - = anion.

In the Type II mechanism PS in the T^1^ state can transfer energy directly to molecular oxygen. This is possible due to the fact that molecular oxygen is also found in the T^1^ conformation in its ground state, thus forming the reactive species in the singlet state, which has strong oxidizing properties, outlined below (Benov, 2015; Tedesco et al., 2017).

Type II Mechanism–Mediated by the production of ^1^O_2_, as an example, lipid peroxidation.

P0S→P1S→P3S

P3S+O23→P0S+O21(energy transfer)

O21+S→S−OOH(peroxides,etc.)

PS = photosensitizer and S = organic substrate.

In both mechanisms, a series of product responses are initiated, causing different effects and biological responses, such as oxidative stress to pathological tissue, and cell damage followed by death (Foot, 1991; Tedesco and Jesus, 2017).

### General Approach to Photosensitizers

PS are one of the three crucial elements of PDT, PS are natural or synthetic molecules capable of absorbing energy and transferring this energy to neighboring molecules (Tedesco et al., 2017). PS have been used to treat disease for over 4,000 years ago. The ancient Egyptians used plants and solar light for vitiligo treatment. However, the advancement of PDT came with the emergence of first-generation PS, the derivatives of hematoporphyrin, its commercial forms called Photofrin, Photosan, Photogen and Photocarcinorin (Sternberg et al., 1998). Although these PS have been widely used in experimental clinical studies, they have some disadvantages, such as low solubility in aqueous media, low selectivity for pathogenic tissues, difficulty in purifying molecules and skin sensitivity (Dobson et al., 2018; Imberti et al., 2020).

These limitations of the PS stimulated the development of the second generation of PS with greater efficiency in ROS generation. The main characteristics of good PS are: high selectivity for pathogenic tissue, high production of singlet oxygen and free radicals, absence of dark toxicity and high absorption in the 600–800 nm wavelength region (Zhang et al., 2018). The second generation PS group is currently composed of hematoporphyrin derivatives, synthetic SF such as 5-aminolevulinic acid, benzoporphyrin derivatives, texaphyrins, thiopurine derivatives, chlorin and phthalocyanines (Agostinis et al., 2011). The 5-aminolevulinic acid (ALA) a precursor of protoporphyrin IX and which works as a pro-drug has become an important discovery for PDT. ALA becomes a PS only after its transformation into protoporphyrin, so this pro-drug can be used in various administration routes such as topical or oral (De Rosa and Bentley, 2000; Morton, 2002). Second-generation PS overcame the disadvantages of first-generation PS, such as greater chemical purity, greater tissue permeation, greater singlet oxygen production, decreased side effects, thus increasing selectivity for pathogenic tissues and faster elimination of PS from the body, however, the main disadvantage of these new PS is their low solubility in aqueous media, which becomes a very limiting factor regarding the administration of these PS, which requires the use of new methods for the delivery of this PS (Kwiatkowski et al., 2018).

The third generation photosensitizer emerged with the interest to improve the selectivity of therapy, this new generation is based on the organic synthesis of new molecules with greater affinity to the pathogenic tissue, in addition to having the objective of expanding the administration routes of these photosensitizers (Kwiatkowski et al., 2018; Quina and Silva, 2021). Has been carried out by combining second-generation photosensitizers with receptor molecules to the desired target, such as proteins or lipoproteins that are used by pathogenic cells for their proliferation, monoclonal antibodies targeting a specific antigen of the target cell, surface markers such as, growth factors, hormones, or transferrin receptors (Muehlmann et al., 2014; Zhang et al., 2018). These strategies allow greater delivery of the photosensitizer to the target tissue, that is, greater selectivity, which improves the effectiveness of PDT, in addition to decreasing the doses needed for desired therapeutic responses (Calixto et al., 2016; Zhou et al., 2021).

## Tissue Engineering and PDT Applications

PDT has mainly emerged as a new alternative treatment to conventional anti-cancer therapies that cause various side effects (Zhang et al., 2018). In the last 3 decades, several types of PS have been applied in pre-clinical and clinical studies (Table 1). In addition to some of these molecules reaching the market and showing great efficacy in the treatment of different types of cancer (Zhang et al., 2018).

**TABLE 1:** Photosensitizers investigated in clinical trial for cancer treatment^a^ (Chen et al., 2001; Pogue et al., 2001; Cramers et al., 2003; Agostinis et al., 2011, 2012; Lamberti et al., 2014; Bacellar et al., 2015; Spring et al., 2016; Banerjee et al., 2017; Kwiatkowski et al., 2018; Dos Santos et al., 2019).

Photosensitizer	Chemical family	Cancer type
Porfirmer sodium, HPD: hematoporphyrin derivative (Photofrin)	Porphyrin	Lung, Esophagus, Bile Duct, Bladder, Brain, Ovarian, Breast Skin Metastases
5-ALA: 5-aminolevulinic acid (Levulan)	Porphyrin Precursor	Skin, Bladder, Brain, Esophagus
MAL: methyl-aminolevulinate (Metvix)	Porphyrin Precursor	Skin
h-ALA: hexylaminolevulinate (Hexvix)	Porphyrin Precursor	Basal Cell
Veteporfin, BDP: benzoporphyrin derivative (Visudyne)	Porphyrin	Pancreas, Breast, Ophthalmic, Skin
Palladium bactereopheophorbide, padeliporfin, WST-11 (Tookad)	Porphyrin	Esophagus, Prostate
Temoporfin, mTHPC: meso-tetrahydroxyphenylchlorine (Foscan)	Chlorin	Head And Neck, Lung, Brain, Bile Duct, Pancreas Skin, Breast
Talaporfin, mono-L-aspartyl chlorin e6, NPe6, LS11 (Laserphyrin)	Chlorin	Liver, Colon, Brain, Lung, Breast Skin Metastases
HPPH: 2-(1-hexyloxyethyl)-2-devinyl pyropheophorbide-a (Photochlor)	Chlorin	Head And Neck, Esophagus, Lung
Rostaporfin, SnEt2: tin ethyl etiopurpurin I, or (Purlytin)	Chlorin	Skin, Breast
Fimaporfin, disulfonated tetraphenyl chlorin, TPCS2a (Amphinex)	Chlorin	Superficial Cancers, Cholon
Motexafin lutetium (Lutex)	Texaphyrin	Breast
Foscan (mTHPC)	Chlorine	Head And Neck, Lung, Brain, Skin, Bile Duct
Purlytin (SnEt2)	Chlorin	Skin, Breast
Taloporfin, LS11, MACE, Npe6	Chlorin	Liver, Colon, Brain
Fotolon (PVP-Ce6), Radachlorin, Photodithazine	Chlorin	Nasopharyngeal, Sarcoma, Brain
Silicon phthalocyanine (PC4)	Phthalocyanine	Cutaneous T Cell Lymphoma
Padoporfin (TOOKAD)	Bacteriochlorin	Prostate

a Adapted from (Agostinis et al., 2012; Dos Santos et al., 2019).

Although the initial focus of the use of PDT was the treatment of several types of cancer, PDT can also be used in the treatment of many other diseases (Yoo et al., 2021). Table 2 presents a summary of some clinical and preclinical studies for non-cancer diseases that use PDT as a treatment.

**TABLE 2:** Photosensitizers investigated in clinical and preclinical trials for non-cancer diseases treatment.

Applications	Diseases	Photosensitizer	References
Dermatologic Disease	acnes, warts, photoaging, psoriasis, vascular malformations, cutaneous leishmaniasis, onychomycosis, hirsutism, keloids, alopecia areata	5-Aminolaevulinic acid (ALA)	Orenstein et al. (1990); Stender et al. (2000); Goldberg, (2008); Jerjes et al. (2011); Calzavara-Pinton et al. (2013); Yang et al. (2013); Almutawa et al. (2015); Zhang et al. (2015); Shin et al. (2015); Zhao et al. (2016); Giorgio et al. (2020); Morton et al. (2020)
Ophthalmologic Disease	Central Serous Chorioretinopathy, Age-Related Macular Degeneration, Corneal Neovascularization	Verteporfin, Indocyanine Green	Díaz-Dávalos et al. (2016); Gao et al. (2018); van Dijk et al. (2018); Boon et al. (2019)
Cardiovascular Disease	Atherosclerosis, Esophageal Varix	5-Aminolaevulinic acid (ALA), Indocyanine Green, Porphirin, Motexafin Lutetium, Chlorine(e6)	Rockson et al. (2000); Tawakol et al. (2006); Li et al. (2009); Qiu et al. (2012); Patel et al. (2013); Houthoofd et al. (2020)
Dental Disease	Periodontitis, Oral Lichen Planus	Curcumin, Indocyanine Green, Phenothiazine, Methylene Blue	De Oliveira et al. (2007); Lavanya et al. (2011); Sadaksharam et al. (2012); Cosgarea et al. (2020); Joshi et al. (2020); Botelho et al. (2021); Pérez-Pacheco et al. (2021)
Neurologic Disease	Alzheimer’s Disease, Prion Disease	Rose Bengal, Methylene Blue, Porphyrin, Phthalocyanine	Murphy, (2002); Cobb and Surewicz, (2009); White and Mallucci, (2009); Benilova et al. (2012); Lee et al. (2015); Lee et al. (2017); Kostelanska et al. (2019)
Skeletal Disease	Rheumatoid Arthritis, Synovitis	Chloroquine, 5-Aminolaevulinic acid (ALA), Phthalocyanine	Hendrich and Siebert, (1997); Kirdaite et al. (2002); Dietze and Berg, (2005); Gallardo-Villagrán et al. (2019)
Gastrointestinal Disease	Crohn’s Disease, Bacteria-Mediated Gastritis or Colitis	5-Aminolaevulinic acid (ALA), Porphirin, Methylene Blue	Cassidy et al. (2011); Baccani et al. (2019)
Respiratory Disease	Ventilator-Associated Pneumonia, COVID-19	Methylene Blue, Curcumin	Almeida et al. (2020); Gendrot et al. (2020); Moghissi et al. (2020); Zangirolami et al. (2020)

There is a lot of effort to use PDT for the treatment of different types of diseases (Yoo et al., 2021). For that reason, it is extremely important to know the PS that has currently been employed in order to verify if there is potential for a new therapeutic application (Kou et al., 2017).

TE is an area in constant expansion and its use in association with PDT has shown promising results in some studies, especially in studies involving antitumor therapy (Table 3).

**TABLE 3:** Overview of studies involving 3D tissue engineering model for application in Photodynamic Therapy.

3D model	Photosensitizer (s)	Photodynamic therapy parameters	Main conclusions	References
Inflammatory breast cancer	Benzoporphyrin derivative monoacid A (BPD) and N-aspartyl chlorin e6 (NPe6)	BPD-PDT: 1.5 µM (BPD) for 60 min	MAME model of IBC were killed at a 45 mJ/cm^2^ BPD–PDT dose. When the light dose was increased, there was a progressive decrease in cell viability	Aggarwal et al. (2015)
		Wavelength: 690 ± 10 nm		
		Post-irradiation: 37°C (18 and 24 h)		
		BPD- NPe6—PDT: 1.5 µM (BPD) and 40 µM (NPe6) for 60 min		
		Light dose: 45–540 mJ/cm^2^	The combination of BPD and NPe6 generated photokilling of IBC MAME structures by apoptosis. This could be seen through the activation of caspase-3 and changes in nuclear morphology	
		Wavelength: 690 and 660 nm		
		Light Source: Halogen light (1.5 mW/cm^2^)		
		Post-irradiation: 37°C (24 and 48 h)		
Micrometastatic pancreatic cancer	Benzoporphyrin derivative (BPD, verteporfin)	BPD-PDT: 0.25 µM (BPD) for 90 min	The use of 3D models with computational analysis of treatment results allows testing a large number of combinations, which are necessary to establish the most effective set of treatment conditions. PDT can be employed as a postoperative procedure to prevent peritoneal carcinomatosis after surgery, for which the current study provides promising preclinical evidence	Broekgaarden et al. (2019)
		Light dose: 1–50 J/cm^2^		
		Wavelength: 690 nm		
		Light Source: Halogen light (50 mW/cm^2^)		
		Post-irradiation: 37°C (24 and 48 h)		
Mesothelioma	Photofrin	Photofrin-erlotinib-PDT: 4 mM (erlotinib solution) and 10 µg/ml (Photofrin) overnight	A new 3D cell culture method for malignant pleural mesothelioma (MPM) was developed. Erlotinib increases the direct cytotoxicity of Photofrin-mediated PDT without altering Photofrin uptake. The 3D model is suitable for further analysis such as flow cytometry. Potential use of receptor tyrosine kinase inhibitors to improve clinical PDT response	Cramer et al. (2019)
		Light dose: 4 J/cm^2^		
		Wavelength: 632 nm		
		Light Source: Red light (light		
		emitting diodes)		
		Post-irradiation: 37°C (24 h)		
Squamous cell carcinoma	5,10,15,20-tetrakis (1-methyl 4-pyridinio) porphyrin tetra (p-toluenesulfonate) (TMPyP)	TMPyP-PDT with or without gold nanorods (Au NRs): 20 μg/mL^1^ and 1,08 μg/mL^1^ for 7 and 20min. Light Source: blue LumiSource^®^ flatbed lamp, peak emission at 420 nm and 7 mW/cm^2^ output. Post-irradiation: 37°C (24 h)	The loading of TMPyP to Au NRs enhances the absorbance and emission intensity of the PS and improves the ROS generation by light irradiation under *in vitro* cell culture conditions. For short-term illumination, showed significantly higher phototoxicity compared to free PS at equivalent concentrations. Au NRs loaded with TMPyP are promising agents for photodynamic therapy and fluorescence imaging of HNSCC.	Demir Duman et al. (2020)
Cervical carcinoma	5,10,15,20-tetra (m-hydroxyphenyl) chlorin (m-THPC - Foscan^®^)	m-THPC-PDT: 0,05, 0,25, 0,1, 0,5 e 2 µg/ml for 3 and 24 h	Viability data indicate that the most effective light source is LED A (violet), followed by LED D (deep red). It is important to emphasize that the results in the present work support the utilization of violet LED light to treat the early stages of neoplastic cervical diseases	Etcheverry et al. (2020)
		Light source: different LED sources (exposure time (tI) of 30, 20, 15, 8, 4, 2, 1 and 0.5 min)		
		A: Emitting range (nm): 390–415; Irradiance (µW/cm^2^): 12.41; Illuminance (lux; lm/m^2^): 11.37; Photon flux (cm^2^): 7.0. B: Emitting range (nm): 440–470; Irradiance (µW/cm^2^): 12.92; Illuminance (lux; lm/m^2^): 234,1; Photon flux (cm^2^): 17.5. C: Emitting range (nm): 620–645; Irradiance (µW/cm^2^): 12.24; Illuminance (lux; lm/m^2^): 467.0; Photon flux (cm^2^): 12.0. D: Emitting range (nm): 640–670; Irradiance (µW/cm^2^): 12.89; Illuminance (lux; lm/m^2^): 173,5; Photon flux (cm^2^): 14.0. Post-irradiation: 37°C (6 and 24 h)		
Pancreatic Cancer	Benzoporphyrin derivative (BPD, verteporfin)	BPD-PDT: 250 nmol/L for 1 h	Coculture with fibroblasts in this case enhanced the PDT response. The high sensitivity of the stromal compartment itself points to the potential of PDT as an adjuvant therapy for stromal depletion, not only priming the tumor for increased death response, as seen here, but also potentially enhanced permeability of the notoriously dense Pancreatic ductal adenocarcinoma (PDAC) stroma to subsequent drug delivery	Karimnia et al. (2021)
		Light source: Laser		
		Wavelength: 690 nm		
		Light Dose: 5–20 J/cm		
		Post-irradiation: 37°C (24 h)		
Spheroidal cell models of colorectal cancer	Hypericin	Hypericin-PDT: 0–200 nM for 16 h. Light source: LED; Wavelength: 594 nm; Dose light: 1 J/cm^2^; Light treatment: 72 min and 28 s at 0.23 mW/cm2. The 3D models were incubated with 10 μM Ko143 at 37 °C/5% CO2/95% for 90 min followed by the addition of increasing doses of Hypericin (0–200 nM) for an additional 16 h	Hypericin-PDT has reduced efficacy in colorectal cancer spheroids as compared to 2D cultures, which may be attributable through upregulation in ABCG2. The clinical efficacy of Hypericin-PDT may be enhanced by ABCG2 inhibition	Khot et al. (2018)
Nasopharyngeal carcinoma	Liposomal formulations of Temoporfin (m-THPC) [3,3′,3″,3”’-(2,3-dihydroporphyrin-5,10,15,20-tetrayl) tetraphenol] (m-THPC- Fospeg^®^)	FosPeg®-PDT: 0.001 μg/ml to 5 μg/ml for 24 h	3D spheroids, especially the method with agarose base (MCL) spheroids, are more suitable for *in vitro* evaluation of FosPeg^®^ mediated PDT effect on nasopharyngeal carcinoma cells. Further study on other photosensitizers are needed to prove the generality of the 3D models developed in this study	Wu et al. (2020a)
		Light source: Laser, Wavelength: 652 nm		
		Light dose:t 5–20 J/cm^2^		
Multicellular tumor spheroids of head and neck squamous cell carcinoma	Temoporfina (mTHPC), Cloro e6 (Ce6) and Indocyanine green (ICG)	mTHPC-Ce6-ICG-PDT: 4,5 and 40 μM	They demonstrated that the presence of stroma influences the behavior of photoactive drugs in different ways: 1°) No effect on Indocyanine Green distribution; 2°) lower accumulation of Chlorin e6; 3°) better penetration and PDT efficiency of Temoporfin. The developed stroma-rich spheroids enlarge the arsenal of *in vitro* pre-clinical models for high-throughput screening of anti-cancer drugs	Yakavets et al. (2019)
		Light source: Red light, 652 nm		
		Light dose: 20 J/cm^2^		

In the study by Cramer et al. (2019) a 3D model of malignant pleural mesothelioma (MPM) was developed to assess the effect of PDT using a 1st generation PS, Photofrin. First, they tested different combinations of scaffolds for cell growth: 1) agarose; 2) agarose-collagen type I; 3) agarose overlay preceded by hanging-drop; 4) GFR-matrigel. They observed that the combination containing collagen provided cell growth on the 7th day. However, some of the cell lines used did not grow under these conditions. The opposite was observed in GFR-Matrigel, cell growth was more efficient and consistent than the other combinations. Therefore, a 3D model containing Matrigel and type I collagen was used to evaluate the effect of PDT-Photofrin. For this, the protocol involved the use of erlotinib, an inhibitor of epidermal growth factor (EGFR) in order to confirm the hypothesis that this inhibition could improve the outcome of PDT. They concluded that the 3D model obtained can be used for future studies, allowing the analysis that the use of erlotinib was able to improve the cytotoxicity of PDT-Photofrin (Cramer et al., 2019).

Second-generation PS has been well described in the literature for clinical studies (Tables 1, 2). In this context, Aggarwal et al. (2015) reported in their work the development of a 3D model of inflammatory breast cancer (IBC MAME) for application in PDT. The IBC MAME model was obtained from reconstituted basement membrane (rBM) and different breast cancer cell lines. After 7 days the formation of structures occurred. Subsequently, experiments involving PDT were conducted using two protocols. In the first protocol, the photosensitizer derived from benzoporphyrin monoacid A (BPD) was used with a light dose ranging from 45 to 540 mJ/cm^2^. For the second protocol, they combined BPD and with PS N-aspartyl chlorin e6 (NPe6) (NPe6/BPD), which were incubated for 60 min and activated sequentially. They observed that the NPe6/BPD-PDT protocol was more efficient in the photo death of tumor cells compared to the first protocol. In addition, the light dose required to obtain death above 90% for the NPe6/BPD-PDT protocol was 45 mJ/cm^2^. Obtaining this same death rate for BPD-PDT required an 8-fold higher light dose (Aggarwal et al., 2015).

Broekgaarden et al. (2019) evaluated the efficiency of PDT using BPD alone and in combination with the chemotherapy drug oxyplatin in a 3D culture model of metastatic pancreatic cancer. 3D culture models were established from different pancreatic cancer cell lines on matrigel scaffolds, which were kept in culture for 18 days. After the eighth day, the PDT assay was conducted. They used in the PDT protocol 0.25 µM of BPD, incubation for 1.5 h, laser light of 690 nm, irradiance of 50 mW/cm^2^ and light dose of 1–50 J/cm^2^, the effects of the treatment were evaluated in the days 9, 11 and 18 post-treatment. For the chemotherapy protocol, oxyplatin was used for 72 h and the treatment effects were analyzed on day 11 and 18. The authors observed through the results that PDT combined with oxyplatin was more efficient than monotherapy. In addition, they noted that the effectiveness of the treatment was time-dependent. They concluded that PDT can prevent peritoneal carcinomatosis after surgery, which for the present study provides promising preclinical evidence (Broekgaarden et al., 2019).

Another study involving a 3D model of the pancreas was developed by Karimnia et al. (2021). The 3D model used presented in its composition co-culture of pancreatic cancer tumor cells and human fibroblasts and matrigel as a scaffold. After 7 days that the model was obtained, treatment with PDT was performed. The protocol involved the use of PS verteporfin (benzoporphyrin derivative monoacid ring A, BPD), incubation for 1 h, irradiation with a laser source of 690 nm, light dose variation from 5 to 20 J/cm^2^ with irradiation of 100 mW/cm^2^. They treated the 3D model with chemotherapeutic agents (gemcitabine and oxyplatin) in order to compare it with the response obtained by PDT after 24 h of treatment. They noted that the presence of fibroblasts in the 3D model promoted chemoresistance. In contrast, the response was increased to PDT when compared to monoculture. They concluded that PDT may be an efficient strategy to overcome tumor-promoting stromal interactions associated with poor therapeutic response in pancreatic cancer (Karimnia et al., 2021).

The generation of spheroids is frequently reported in the literature as an approach for evaluating the efficacy of drugs *in vitro*. This 3D model has advantages such as being relatively inexpensive with the possibility of co-culture (Gong et al., 2015; Khot et al., 2018; Yakavets et al., 2019; Brancato et al., 2020).

In this context, the surface of the culture plate used to obtain the spheroid plays an important role, mainly in the orientation of cellular behavior (Brancato et al., 2020). Therefore, some authors described in their work the use of agarose coated plate to obtain spheroids (Khot et al., 2018; Cramer et al., 2019; Yakavets et al., 2019).

Multicellular colorectal cancer spheroids to verify the effect of PDT-Hypericin compared to monolayer model was investigated by Khot et al. (2018). The authors concluded that the PDT-Hypericin effect was greater in the 2D culture than in the spheroids. However, using an ABCG2 protein inhibitor caused an increase in the effect of PDT-Hypericin (Khot et al., 2018). Yakavets et al. (2019) produced multicellular head and neck squamous cell carcinoma spheroids in co-culture. As in the work by Khot et al. (2018), the plate pre-coated with agarose to obtain the spheroid was used (Yakavets et al. (2019).

The PDT protocol employed by Yakavets et al. (2019) was based on the use of three second-generation PS, indocyanine green (ICG), temoporphyrin (mTHPC), and Chlorin e6 (Ce6) in co-culture spheroids compared to homospheroids. The authors concluded that tumor stromal components may limit the antitumor activity of anticancer therapies. In the case of the PS used, they observed that Ce6 had less accumulation in the co-culture spheroids, unlike mTHPC, whereas ICG accumulated equally in the two spheroid models compared (Yakavets et al., 2019).

In the data presented in Table 1, it was possible to observe that in the clinical trials for the treatment of cancer most of the PS used belong to the first and second generation. Therefore, 3D model studies involving 3rd generation PS for PDT are essential to enable the expansion of its clinical use (Chen et al., 2001; Pogue et al., 2001; Cramer et al., 2019; Agostinis et al., 2011, 2012; Lamberti et al., 2014; Bacellar et al., 2015; Spring et al., 2016; Banerjee et al., 2017; Kwiatkowski et al., 2018; Dos Santos et al. al., 2019).

In the studies by Demir Duman et al. (2020), Etcheverry et al. (2020) and Wu et al. (2020) the effects of PDT with different third-generation PS on different 3D tumor models were studied. These being 1) nanocomposites of gold nanorods (Au NRs) with the cationic porphyrin TMPyP (5,10,15,20-tetrakis (1-methyl 4-pyridinium)porphyrin tetra (p-toluenesulfonate); 2) m-THPC and 3) pegylated liposomes containing mTHPC, respectively. Results and protocols were varied. There was a consensus that PDT was efficient and further studies on other photosensitizers are needed to prove the generality of the 3D models developed in the studies described (Demir Duman et al., 2020; Etcheverry et al., 2020; Wu et al. (2020).

## Conclusion

Therefore, the association of Tissue Engineering and Photodynamic Therapy protocols resulted in great advances for the understanding of therapeutic processes based on the interest in the interaction of monochromatic light with biological tissues. Tissue Engineering is a field of science in full expansion and would also contribute to a better understanding of photodynamic mechanisms. This scientific review article can directly contribute to the organization of what is considered a state of the art in this field of knowledge. Updating and presenting important information for the direction of works that wish to use these advanced protocols. There is no doubt about the great potential for using these combined concepts, which are at the Frontier of knowledge and can help in the development of new biological assays available for application in various clinical treatments and chronic pathologies such as antitumor, anticancer and chronic psoriasis.

## Challenges and Future Perspectives

The development of tissue engineering has been described in the literature for many decades. In recent years it has gained evidence mainly through the appeal to use alternatives to animal testing. Until now, the studies have involved non-systemic evaluation of drug behavior in human cells/tissues, replacement of damaged parts of the body, cosmetic testing, among others (Langer and Vacanti, 1993; Morales, 2008; Doke and Dhawale, 2015).

However, the biggest challenge related to this technology is to obtain models that faithfully emulate all the characteristics of the human biological structure. In addition, the pathophysiology of certain diseases often has different expressions between species, which becomes another limitation for reproducing the methodologies (Guiro and Arinzeh, 2015; Hapach et al., 2019). Other possible obstacles to the development and application of 3D models mainly involve cell types, as it is necessary to use at least 2 cell types to be able to recreate the original structure and production costs (Langer and Vacanti, 2016).

Despite the challenges, the production of 3D Cell Culture Models is an excellent tool to assess the possibility of transposing data directly to humans (Ravi et al., 2015; Ceylan and Bölgen, 2016). In this context, there are several types of photosensitizing agents available for use in PDT that require biological models to be tested (Desmet et al., 2018; Yakavets et al., 2019; Etcheverry et al., 2020). However, the challenges associated with the structural characteristics of PS need to be overcome for application in PDT and, consequently, make its clinical use unfeasible. They are usually molecules of high molecular weight and lipophilicity, such as porphyrins, which lead to low permeability and make it difficult to incorporate it into conventional pharmaceutical forms (Webber, 2016).

Another factor to be considered for the application of PDT includes the low selectivity of PS action. Therefore, this contributes to the use of nanotechnology (Sharma et al., 2017). Therefore, a strategy to overcome these limitations associated with PS would be the use of delivery systems such as polymeric nanoparticles and liposomes (Sharma et al., 2017).

Therefore, the evolution of TE is related to the possibility of obtaining new tools such as the implantation of biofabricated tissues, elaboration of synthetic scaffolds capable of simulating the tissue’s microenvironment, production of mini-organs, valves, cartilages, among others from 3D bioprinting (Nguyen and Pentoney, 2017; W et al., 2017; Tarassoli et al., 2018). And more recently, the concepts of organ-in-a-chip and human body-on-chip were introduced, which are small three-dimensional biomimetic systems that aim to mimic characteristics of the organs they represent (Low et al.; Chen et al., 2021). In addition to being interconnected to form larger systems with different types of cells on which physical forces act, they have several applications such as analysis of pharmacokinetic, pharmacodynamic and toxicological properties of drugs, organ-organ interaction (Low et al., 2021; Chen et al., 2021).

## References

[B1] Aggarwal N. Santiago A. M. Kessel D. Sloane B. F. (2015). Photodynamic Therapy as an Effective Therapeutic Approach in MAME Models of Inflammatory Breast Cancer. Breast Cancer Res. Treat. 154, 251–262. 10.1007/s10549-015-3618-6 26502410 PMC4753063

[B2] Agostinis P. Berg K. Cengel K. A. Foster T. H. Girotti A. W. Gollnick S. O. (2012). NIH Public Access 61, 250–281. 10.3322/caac.20114 PMC3209659 21617154

[B3] Agostinis P. Berg K. Cengel K. A. Foster T. H. Girotti A. W. Gollnick S. O. (2011). Photodynamic Therapy of Cancer: An Update. CA A Cancer J. Clin. 61, 250–281. 10.3322/caac.20114 PMC3209659 21617154

[B4] Al-Himdani S. Jessop Z. M. Al-Sabah A. Combellack E. Ibrahim A. Doak S. H. (2017). Tissue-Engineered Solutions in Plastic and Reconstructive Surgery: Principles and Practice. Front. Surg. 4, 4. 10.3389/fsurg.2017.00004 28280722 PMC5322281

[B5] Alemany-Ribes M. García-Díaz M. Busom M. Nonell S. Semino C. E. (2013). Toward a 3D Cellular Model for Studying *In Vitro* the Outcome of Photodynamic Treatments: Accounting for the Effects of Tissue Complexity. Tissue Eng. Part A 19, 1665–1674. 10.1089/ten.tea.2012.0661 23442191 PMC3700089

[B6] Almeida A. Faustino M. A. F. Neves M. G. P. M. S. (2020). Antimicrobial Photodynamic Therapy in the Control of COVID-19. Antibiotics 9, 320. 10.3390/antibiotics9060320 PMC7344747 32545171

[B7] Almutawa F. Thalib L. Hekman D. Sun Q. Hamzavi I. Lim H. W. (2015). Efficacy of Localized Phototherapy and Photodynamic Therapy for Psoriasis: A Systematic Review and Meta-Analysis. Photodermatol. Photoimmunol. Photomed. 31, 5–14. 10.1111/phpp.12092 24283358

[B8] Asadi N. Del Bakhshayesh A. R. Davaran S. Akbarzadeh A. (2020). Common Biocompatible Polymeric Materials for Tissue Engineering and Regenerative Medicine. Mater. Chem. Phys. 242, 122528. 10.1016/j.matchemphys.2019.122528

[B9] Baccani I. Faraoni P. Marini M. Gnerucci A. Orsini B. Pecile P. (2019). Synergistic Effect of Photodynamic Therapy at 400 Nm and Doxycycline against *Helicobacter pylori* . Future Microbiol. 14, 1199–1205. 10.2217/fmb-2019-0129 31625444

[B10] Bacellar I. Tsubone T. Pavani C. Baptista M. (2015). Photodynamic Efficiency: From Molecular Photochemistry to Cell Death. Ijms 16, 20523–20559. 10.3390/ijms160920523 26334268 PMC4613217

[B11] Banerjee S. M. MacRobert A. J. Mosse C. A. Periera B. Bown S. G. Keshtgar M. R. S. (2017). Photodynamic Therapy: Inception to Application in Breast Cancer. Breast 31, 105–113. 10.1016/j.breast.2016.09.016 27833041

[B12] Bell E. Ehrlich H. P. Buttle D. J. Nakatsuji T. (1981). Living Tissue Formed *In Vitro* and Accepted as Skin-Equivalent Tissue of Full Thickness. Science 211, 1052–1054. 10.1126/science.7008197 7008197

[B13] Benilova I. Karran E. De Strooper B. (2012). The Toxic Aβ Oligomer and Alzheimer's Disease: an Emperor in Need of Clothes. Nat. Neurosci. 15, 349–357. 10.1038/nn.3028 22286176

[B14] Benov L. (2015). Photodynamic Therapy: Current Status and Future Directions. Med. Princ. Pract. 24, 14–28. 10.1159/000362416 24820409 PMC6489067

[B16] Blum K. M. Mirhaidari G. J. M. Breuer C. K. (2022). Tissue Engineering: Relevance to Neonatal Congenital Heart Disease. Seminars Fetal Neonatal Med. 27, 101225. 10.1016/j.siny.2021.101225 PMC8390581 33674254

[B17] Bodnar C. Christiani T. R. Dahm K. Vernengo A. J. (2018). Implementation and Assessment of an Undergraduate Tissue Engineering Laboratory Course. Educ. Chem. Eng. 24, 52–59. 10.1016/j.ece.2018.07.002

[B18] Boon C. J. F. Van Dijk E. H. C. Yzer S. (2019). Photodynamic Therapy in Central Serous Chorioretinopathy. Cent. Serous Chorioretinopathy 28, 283–292. 10.1016/B978-0-12-816800-4.00019-X

[B19] Botelho J. Machado V. Hussain S. B. Zehra S. A. Proença L. Orlandi M. (2021). Periodontitis and Circulating Blood Cell Profiles: a Systematic Review and Meta-Analysis. Exp. Hematol. 93, 1–13. 10.1016/j.exphem.2020.10.001 33068648

[B20] Bourland J. Fradette J. Auger F. A. (2018). Tissue-engineered 3D Melanoma Model with Blood and Lymphatic Capillaries for Drug Development. Sci. Rep. 8. 10.1038/s41598-018-31502-6 PMC6123405 30181613

[B21] Brancato V. Oliveira J. M. Correlo V. M. Reis R. L. Kundu S. C. (2020). Could 3D Models of Cancer Enhance Drug Screening? Biomaterials 232, 119744. 10.1016/j.biomaterials.2019.119744 31918229

[B22] Broekgaarden M. Bulin A.-L. Frederick J. Mai Z. Hasan T. (2019). Tracking Photodynamic- and Chemotherapy-Induced Redox-State Perturbations in 3D Culture Models of Pancreatic Cancer: A Tool for Identifying Therapy-Induced Metabolic Changes. Jcm 8, 1399. 10.3390/jcm8091399 PMC6788194 31500115

[B23] Calixto G. Bernegossi J. De Freitas L. Fontana C. Chorilli M. Grumezescu A. M. (2016). Nanotechnology-based Drug Delivery Systems for Photodynamic Therapy of Cancer: A Review. Molecules 21, 342. 10.3390/molecules21030342 26978341 PMC6274468

[B24] Calzavara-Pinton P. G. Rossi M. T. Aronson E. Sala R. The Italian Group for Photodynamic Therapy N. Burtica E. C. (2013). A Retrospective Analysis of Real-Life Practice of Off-Label Photodynamic Therapy Using Methyl Aminolevulinate (MAL-PDT) in 20 Italian Dermatology Departments. Part 1: Inflammatory and Aesthetic Indications. Photochem. Photobiol. Sci. 12, 148–157. 10.1039/c2pp25124h 22949035

[B25] Cassidy C. M. Tunney M. M. Caldwell D. L. Andrews G. P. Donnelly R. F. (2011). Development of Novel Oral Formulations Prepared via Hot Melt Extrusion for Targeted Delivery of Photosensitizer to the Colon. Photochem. Photobiol. 87, 867–876. 10.1111/j.1751-1097.2011.00915.x 21375536

[B26] Ceylan R. Kiefer M. (2016). Ausblick und Thesen. Biomater. Biomech. Bioeng. 3, 141–147. 10.1007/978-3-658-10583-9_5

[B27] Chen X. Zhang Y. S. Zhang X. Liu C. (2021). Organ-on-a-chip Platforms for Accelerating the Evaluation of Nanomedicine. Bioact. Mater. 6, 1012–1027. 10.1016/j.bioactmat.2020.09.022 33102943 PMC7566214

[B28] Chen Z. Woodburn K. W. Shi C. Adelman D. C. Rogers C. Simon D. I. (2001). Photodynamic Therapy with Motexafin Lutetium Induces Redox-Sensitive Apoptosis of Vascular Cells. Atvb 21, 759–764. 10.1161/01.ATV.21.5.759 11348871

[B29] Cobb N. J. Surewicz W. K. (2009). Prion Diseases and Their Biochemical Mechanisms. Biochemistry 48, 2574–2585. 10.1021/bi900108v 19239250 PMC2805067

[B30] Colley H. E. Hearnden V. Jones A. V. Weinreb P. H. Violette S. M. MacNeil S. (2011). Development of Tissue-Engineered Models of Oral Dysplasia and Early Invasive Oral Squamous Cell Carcinoma. Br. J. Cancer 105, 1582–1592. 10.1038/bjc.2011.403 21989184 PMC3242522

[B31] Cosgarea R. Pollmann R. Sharif J. Schmidt T. Stein R. Bodea A. (2020). Photodynamic Therapy in Oral Lichen Planus: A Prospective Case-Controlled Pilot Study. Sci. Rep. 10, 1667. 10.1038/s41598-020-58548-9 32015380 PMC6997407

[B32] Cramer G. Shin M. Hagan S. Katz S. I. Simone C. B. Busch T. M. (2019). Modeling Epidermal Growth Factor Inhibitor-Mediated Enhancement of Photodynamic Therapy Efficacy Using 3D Mesothelioma Cell Culture. Photochem. Photobiol. 95, 397–405. 10.1111/php.13067 30499112 PMC6553615

[B33] Cramers P. Ruevekamp M. Oppelaar H. Dalesio O. Baas P. Stewart F. A. (2003). Foscan Uptake and Tissue Distribution in Relation to Photodynamic Efficacy. Br. J. Cancer 88, 283–290. 10.1038/sj.bjc.6600682 12610515 PMC2377038

[B34] Dai T. Fuchs B. B. Coleman J. J. Prates R. A. Astrakas C. St. Denis T. G. (2012). Concepts and Principles of Photodynamic Therapy as an Alternative Antifungal Discovery Platform. Front. Microbio. 3, 1–16. 10.3389/fmicb.2012.00120 PMC3322354 22514547

[B35] De Oliveira R. R. Schwartz-Filho H. O. Novaes A. B. Taba M. (2007). Antimicrobial Photodynamic Therapy in the Non-surgical Treatment of Aggressive Periodontitis: A Preliminary Randomized Controlled Clinical Study. J. Periodontology 78, 965–973. 10.1902/jop.2007.060494 17539707

[B36] De Rosa F. S. Bentley M. V. L. B. (2000). Photodynamic Therapy of Skin Cancers: Sensitizers, Clinical Studies and Future Directives. Pharm. Res. 17, 1447–1455. 10.1023/A:1007612905378 11303952

[B37] Demir Duman F. Sebek M. Thanh N. T. K. Loizidou M. Shakib K. MacRobert A. J. (2020). Enhanced Photodynamic Therapy and Fluorescence Imaging Using Gold Nanorods for Porphyrin Delivery in a Novel *In Vitro* Squamous Cell Carcinoma 3D Model. J. Mat. Chem. B 8, 5131–5142. 10.1039/d0tb00810a 32420578

[B38] Díaz-Dávalos C. D. Carrasco-Quiroz A. Rivera-Díez D. (2016). Neovascularization Corneal Regression in Patients Treated with Photodynamic Therapy with Verteporfin. Rev. Med. Inst. Mex. Seguro Soc. 54, 164–169. 26960043

[B39] Dietze A. Berg K. (2005). ALA-induced Porphyrin Formation and Fluorescence in Synovitis Tissue. Photodiagnosis Photodyn. Ther. 2, 299–307. 10.1016/S1572-1000(05)00107-9 25048871

[B40] Dobson J. de Queiroz G. F. Golding J. P. (2018). Photodynamic Therapy and Diagnosis: Principles and Comparative Aspects. Veterinary J. 233, 8–18. 10.1016/j.tvjl.2017.11.012 29486883

[B41] Doke S. K. Dhawale S. C. (2015). Alternatives to Animal Testing: A Review. Saudi Pharm. J. 23, 223–229. 10.1016/j.jsps.2013.11.002 26106269 PMC4475840

[B42] Dos Santos A. l. F. De Almeida D. R. Q. Terra L. F. Baptista M. c. S. Labriola L. (2019). Photodynamic Therapy in Cancer Treatment - an Update Review. J. Cancer Metastasis Treat. 2019. 10.20517/2394-4722.2018.83

[B43] Etcheverry M. E. Pasquale M. A. Bergna C. Ponzinibbio C. Garavaglia M. (2020). Photodynamic Therapy in 2D and 3D Human Cervical Carcinoma Cell Cultures Employing LED Light Sources Emitting at Different Wavelengths. Phys. Med. Biol. 65, 015017. 10.1088/1361-6560/ab589a 31739296

[B44] Fitzgerald K. A. Malhotra M. Curtin C. M. O' Brien F. J. O' Driscoll C. M. (2015). Life in 3D Is Never Flat: 3D Models to Optimise Drug Delivery. J. Control. Release 215, 39–54. 10.1016/j.jconrel.2015.07.020 26220617

[B45] Foote C. S. (1991). Definition of Type I and Type II Photosensitized Oxidation. Photochem. Photobiol. 54, 659. 10.1111/j.1751-1097.1991.tb02071.x 1798741

[B46] Gallardo-Villagrán M. Leger D. Y. Liagre B. Therrien B. (2019). Photosensitizers Used in the Photodynamic Therapy of Rheumatoid Arthritis. Int. J. Mol. Sci. 20, 3339. 10.3390/ijms20133339 PMC6651633 31284664

[B47] Gao Y. Yu T. Zhang Y. Dang G. (2018). Anti-VEGF Monotherapy versus Photodynamic Therapy and Anti-vegf Combination Treatment for Neovascular Age-Related Macular Degeneration: A Meta-Analysis. Investig. Ophthalmol. Vis. Sci. 59, 4307–4317. 10.1167/iovs.17-23747 30372759

[B48] Gendrot M. Andreani J. Duflot I. Boxberger M. Le Bideau M. Mosnier J. (2020). Methylene Blue Inhibits Replication of SARS-CoV-2 *In Vitro* . Int. J. Antimicrob. Agents 56, 106202. 10.1016/j.ijantimicag.2020.106202 33075512 PMC7566888

[B49] Giorgio C. M. Babino G. Caccavale S. Russo T. De Rosa A. B. Alfano R. (2020). Combination of Photodynamic Therapy with 5‐aminolaevulinic Acid and Microneedling in the Treatment of Alopecia Areata Resistant to Conventional Therapies: Our Experience with 41 Patients. Clin. Exp. Dermatol. 45, 323–326. 10.1111/ced.14084 31469914

[B50] Goldberg D. J. (2008). Photodynamic Therapy in Skin Rejuvenation. Clin. Dermatology 26, 608–613. 10.1016/j.clindermatol.2007.09.009 18940541

[B51] Gong X. Lin C. Cheng J. Su J. Zhao H. Liu T. (2015). Generation of Multicellular Tumor Spheroids with Microwell-Based Agarose Scaffolds for Drug Testing. PloS one 10 (6), e0130348. 10.1371/journal.pone.0130348 26090664 PMC4474551

[B52] Grifno G. N. Farrell A. M. Linville R. M. Arevalo D. Kim J. H. Gu L. (2019). Tissue-engineered Blood-Brain Barrier Models via Directed Differentiation of Human Induced Pluripotent Stem Cells. Sci. Rep. 9, 1–13. 10.1038/s41598-019-50193-1 31562392 PMC6764995

[B53] Groeber F. Engelhardt L. Lange J. Kurdyn S. Schmid F. F. Rücker C. (2016). A First Vascularized Skin Equivalent for as an Alternative to Animal Experimentation. ALTEX 33, 415–422. 10.14573/altex.1604041 27180196

[B54] Guiro K. Arinzeh T. L. (2015). Bioengineering Models for Breast Cancer Research. Breast Cancer (Auckl) 9 (Suppl. 2), 57–70. 10.4137/BCBCR.S29424 26792996 PMC4712981

[B55] Hapach L. A. Mosier J. A. Wang W. Reinhart-King C. A. (2019). Engineered Models to Parse Apart the Metastatic Cascade. npj Precis. Onc. 3. 10.1038/s41698-019-0092-3 PMC6704099 31453371

[B56] Hasan A. Morshed M. Memic A. Hassan S. Webster T. Marei H. (2018). Nanoparticles in Tissue Engineering: Applications, Challenges and Prospects. Int. J. Nanomedicine 13, 5637–5655. 10.2147/IJN.S153758 30288038 PMC6161712

[B57] Hendrich C. Siebert W. E. (1997). Photodynamic Therapy for Rheumatoid Arthritis? Lasers Surg. Med. 21, 359–364. 10.1002/(SICI)1096-9101(1997)21:4<359::AID-LSM7>3.0.CO;2-P 9328983

[B58] Herreros-Pomares A. Zhou X. Calabuig-Fariñas S. Lee S.-J. Torres S. Esworthy T.. Hann S. Y. Jantus-Lewintre E. Camps C. Zhang L. G. (2021). 3D Printing Novel *In Vitro* Cancer Cell Culture Model Systems for Lung Cancer Stem Cell Study. Mater. Sci. Eng. C 122, 111914. 10.1016/j.msec.2021.111914 33641907

[B59] Houthoofd S. Vuylsteke M. Mordon S. Fourneau I. (2020). Photodynamic Therapy for Atherosclerosis. The Potential of Indocyanine Green. Photodiagnosis Photodyn. Ther. 29, 101568. 10.1016/j.pdpdt.2019.10.003 31627015

[B60] Imberti C. Zhang P. Huang H. Sadler P. J. (2020). New Designs for Phototherapeutic Transition Metal Complexes. Angew. Chem. Int. Ed. 59, 61–73. 10.1002/anie.201905171 PMC6973108 31310436

[B61] Jerjes W. Upile T. Hamdoon Z. Mosse C. A. Akram S. Morley S. (2011). Interstitial PDT for Vascular Anomalies. Lasers Surg. Med. 43, 357–365. 10.1002/lsm.21058 21674540

[B62] Joshi K. Baiju C. S. Khashu H. Bansal S. (2020). Clinical Effectiveness of Indocyanine Green Mediated Antimicrobial Photodynamic Therapy as an Adjunct to Scaling Root Planing in Treatment of Chronic Periodontitis- A Randomized Controlled Clinical Trial. Photodiagnosis Photodyn. Ther. 29, 101591. 10.1016/j.pdpdt.2019.101591 31783161

[B63] Karimnia V. Rizvi I. Slack F. J. Celli J. P. (2021). Photodestruction of Stromal Fibroblasts Enhances Tumor Response to PDT in 3D Pancreatic Cancer Coculture Models. Photochem. Photobiol. 97, 416–426. 10.1111/php.13339 33011973 PMC7965253

[B64] Kessel D. (2019). Photodynamic Therapy: A Brief History. J. Clin. Med. 8, 1581. 10.3390/jcm8101581 PMC6832404 31581613

[B65] Khot M. I. Perry S. L. Maisey T. Armstrong G. Andrew H. Hughes T. A. (2018). Inhibiting ABCG2 Could Potentially Enhance the Efficacy of Hypericin-Mediated Photodynamic Therapy in Spheroidal Cell Models of Colorectal Cancer. Photodiagnosis Photodyn. Ther. 23, 221–229. 10.1016/j.pdpdt.2018.06.027 29969677

[B66] Kirdaite G. Lange N. Busso N. Van Den Bergh H. Kucera P. So A. (2002). Protoporphyrin IX Photodynamic Therapy for Synovitis. Arthritis & Rheumatism 46, 1371–1378. 10.1002/art.10199 12115245

[B67] Kostelanska M. Freisleben J. Backovska Hanusova Z. Mosko T. Vik R. Moravcova D. (2019). Optimization of the Photodynamic Inactivation of Prions by a Phthalocyanine Photosensitizer: The Crucial Involvement of Singlet Oxygen. J. Biophot. 12, 1–13. 10.1002/jbio.201800430 30989822

[B68] Kou J. Dou D. Yang L. (2017). Porphyrin Photosensitizers in Photodynamic Therapy and its Applications. Oncotarget 8 (46), 81591–81603. 10.18632/oncotarget.20189 29113417 PMC5655312

[B69] Kwiatkowski S. Knap B. Przystupski D. Saczko J. Kędzierska E. Knap-Czop K. (2018). Photodynamic Therapy - Mechanisms, Photosensitizers and Combinations. Biomed. Pharmacother. 106, 1098–1107. 10.1016/j.biopha.2018.07.049 30119176

[B70] Lamberti M. J. Rumie Vittar N. B. Rivarola V. A. (2014). Breast Cancer as Photodynamic Therapy Target: Enhanced Therapeutic Efficiency by Overview of Tumor Complexity. World J. Clin. Oncol. 5, 901–907. 10.5306/wjco.v5.i5.901 25493228 PMC4259952

[B71] Langer R. Vacanti J. (2016). Advances in Tissue Engineering. J. Pediatr. Surg. 51, 8–12. 10.1016/j.jpedsurg.2015.10.022 26711689 PMC4733916

[B72] Langer R. Vacanti J. P. (1993). Tissue Engineering. Science 260, 920–926. 10.1126/SCIENCE.8493529 8493529

[B73] Lanza R. Langer R. Vacanti J. P. Atala A. (2020). Principles of Tissue Engineering. Amsterdam, Netherlands: Elsevier. 10.1016/C2018-0-03818-9

[B74] Lavanya N. Rao U. Jayanthi P. Ranganathan K. (2011). Oral Lichen Planus: An Update on Pathogenesis and Treatment. J. Oral Maxillofac. Pathol. 15, 127–132. 10.4103/0973-029X.84474 22529568 PMC3329692

[B75] Lee B. I. Suh Y. S. Chung Y. J. Yu K. Park C. B. (2017). Shedding Light on Alzheimer's β-Amyloidosis: Photosensitized Methylene Blue Inhibits Self-Assembly of β-Amyloid Peptides and Disintegrates Their Aggregates. Sci. Rep. 7, 1–10. 10.1038/s41598-017-07581-2 28790398 PMC5548810

[B76] Lee J. S. Lee B. I. Park C. B. (2015). Photo-induced Inhibition of Alzheimer's β-amyloid Aggregation *In Vitro* by Rose Bengal. Biomaterials 38, 43–49. 10.1016/j.biomaterials.2014.10.058 25457982

[B77] Li C. Z. Cheng L. F. Wang Z. Q. Gu Y. (2009). Attempt of Photodynamic Therapy on Esophageal Varices. Lasers Med. Sci. 24, 167–171. 10.1007/s10103-008-0542-6 18270762

[B78] Li W. Xie Q. Lai L. Mo Z. Peng X. Leng E. (2017). *In Vitro* evaluation of Ruthenium Complexes for Photodynamic Therapy. Photodiagnosis Photodyn. Ther. 18, 83–94. 10.1016/j.pdpdt.2017.02.001 28193566

[B79] Low L. A. Mummery C. Berridge B. R. Austin C. P. Tagle D. A. (2021). Organs-on-chips: into the Next Decade. Nat. Rev. Drug Discov. 20, 345–361. 10.1038/s41573-020-0079-3 32913334

[B80] Luo D. Carter K. A. Miranda D. Lovell J. F. (2017). Chemophototherapy: An Emerging Treatment Option for Solid Tumors. Adv. Sci. 4, 1600106–1600124. 10.1002/advs.201600106 PMC5238751 28105389

[B81] Lv D. Hu Z. Lu L. Lu H. Xu X. (2017). Three-dimensional C-ell C-ulture: A P-owerful T-ool in T-umor R-esearch and D-rug D-iscovery (Review). Oncol. Lett. 14, 6999–7010. 10.3892/ol.2017.7134 29344128 PMC5754907

[B82] Magdeldin T. López-Dávila V. Pape J. Cameron G. W. W. Emberton M. Loizidou M. (2017). Engineering a Vascularised 3D *In Vitro* Model of Cancer Progression. Sci. Rep. 7, 1–9. 10.1038/srep44045 28276469 PMC5343474

[B83] Maheshwari N. Tekade M. Chourasiya Y. Sharma M. C. Deb P. K. Tekade R. K. (2019). Nanotechnology in Tissue Engineering. Cambridge, MA, USA: Academic Press, 225–261. 10.1016/B978-0-12-814427-5.00007-X

[B84] Melville J. C. Mañón V. A. Blackburn C. Young S. (2019). Current Methods of Maxillofacial Tissue Engineering. Oral Maxillofac. Surg. Clin. N. Am. 31, 579–591. 10.1016/j.coms.2019.07.003 31445759

[B85] Moghissi K. Dixon K. Gibbins S. (2020). Does PDT Have Potential in the Treatment of COVID 19 Patients? Photodiagnosis Photodyn. Ther. 31, 101889. 10.1016/j.pdpdt.2020.101889 32592911 PMC7313506

[B86] Mohammad-Hadi L. MacRobert A. J. Loizidou M. Yaghini E. (2018). Photodynamic Therapy in 3D Cancer Models and the Utilisation of Nanodelivery Systems. Nanoscale 10, 1570–1581. 10.1039/c7nr07739d 29308480

[B87] Morales M. M. (2008). Métodos Alternativos à Utilização de Animais Em Pesquisa Científica: Mito Ou Realidade? Ciência Cult. 60 (2), 33–36.

[B88] Morton C. A. Szeimies R. M. Basset‐Séguin N. Calzavara‐Pinton P. G. Gilaberte Y. Hædersdal M. (2020). European Dermatology Forum Guidelines on Topical Photodynamic Therapy 2019 Part 2: Emerging Indications - Field Cancerization, Photorejuvenation and Inflammatory/infective Dermatoses. J. Eur. Acad. Dermatol Venereol. 34, 17–29. 10.1111/jdv.16044 31805604

[B89] Morton C. (2002). The Emerging Role of 5-ALA-PDT in Dermatology: Is PDT Superior to Standard Treatments? J. Dermatological Treat. 13, s25–s29. 10.1080/095466302317414672 12060514

[B90] Muehlmann L. Ma B. Longo J. Santos M. Azevedo R. (2014). Aluminum&ndash;phthalocyanine Chloride Associated to Poly(methyl Vinyl Ether-Co-Maleic Anhydride) Nanoparticles as a New Third-Generation Photosensitizer for Anticancer Photodynamic Therapy. Int. J. Nanomedicine 9, 1199–1213. 10.2147/IJN.S57420 24634582 PMC3952896

[B91] Murphy R. M. (2002). Peptide Aggregation in Neurodegenerative Disease. Annu. Rev. Biomed. Eng. 4, 155–174. 10.1146/annurev.bioeng.4.092801.094202 12117755

[B92] Naahidi S. Jafari M. Logan M. Wang Y. Yuan Y. Bae H. (2017). Biocompatibility of Hydrogel-Based Scaffolds for Tissue Engineering Applications. Biotechnol. Adv. 35, 530–544. 10.1016/j.biotechadv.2017.05.006 28558979

[B94] Nguyen D. G. Pentoney S. L. (2017). Bioprinted Three Dimensional Human Tissues for Toxicology and Disease Modeling. Drug Discov. Today Technol. 23, 37–44. 10.1016/J.DDTEC.2017.03.001 28647084

[B95] Nishiguchi A. Matsusaki M. Kano M. R. Nishihara H. Okano D. Asano Y. (2018). *In Vitro* 3D Blood/lymph-Vascularized Human Stromal Tissues for Preclinical Assays of Cancer Metastasis. Biomaterials 179, 144–155. 10.1016/j.biomaterials.2018.06.019 29986232

[B96] Orenstein A. Nelson J. S. Liaw L.-H. L. Kaplan R. Kimel S. Berns M. W. (1990). Photochemotherapy of Hypervascular Dermal Lesions: A Possible Alternative to Photothermal Therapy? Lasers Surg. Med. 10, 334–343. 10.1002/lsm.1900100406 2144032

[B97] Pal M. Chen H. Lee B. H. Lee J. Y. H. Yip Y. S. Tan N. S. (2019). Epithelial-mesenchymal Transition of Cancer Cells Using Bioengineered Hybrid Scaffold Composed of hydrogel/3D-Fibrous Framework. Sci. Rep. 9, 1–11. 10.1038/s41598-019-45384-9 31222037 PMC6586872

[B98] Patel B. Han E. Swan K. (2013). Richard Schatzki: A Familiar Ring. Am. J. Roentgenol. 201, W678–W682. 10.2214/AJR.13.10748 24147496

[B100] Pérez-Pacheco C. G. Fernandes N. A. R. Primo F. L. Tedesco A. C. Bellile E. Retamal-Valdes B. (2021). Local Application of Curcumin-Loaded Nanoparticles as an Adjunct to Scaling and Root Planing in Periodontitis: Randomized, Placebo-Controlled, Double-Blind Split-Mouth Clinical Trial. Clin. Oral Investig. 25, 3217–3227. 10.1007/s00784-020-03652-3 33125518

[B101] Pogue B. W. Ortel B. Chen N. Redmond R. W. Hasan T. (2001). A Photobiological and Photophysical-Based Study of Phototoxicity of Two Chlorins. Cancer Res. 61, 717–724. 11212274

[B102] Qidwai A. AnnuNabi B. Nabi B. Kotta S. Narang J. K. Baboota S. (2020). Role of Nanocarriers in Photodynamic Therapy. Photodiagnosis Photodyn. Ther. 30, 101782. 10.1016/j.pdpdt.2020.101782 32330611

[B138] Qiu H. Mao Y. Gu Y. Wang Y. Zhu J. Zeng J. (2012). Vascular Targeted Photodynamic Therapy for Bleeding Gastrointestinal Mucosal Vascular Lesions: A Preliminary Study. Photodiagnosis Photodyn. Ther. 9 (2), 109–117. 10.1016/j.pdpdt.2011.11.003 22594980

[B103] Quina F. H. Silva G. T. M. (2021). The Photophysics of Photosensitization: A Brief Overview. J. Photochem. Photobiol. 7, 100042. 10.1016/j.jpap.2021.100042

[B104] Ravi M. Paramesh V. Kaviya S. R. Anuradha E. Solomon F. D. P. (2015). 3D Cell Culture Systems: Advantages and Applications. J. Cell. Physiol. 230, 16–26. 10.1002/jcp.24683 24912145

[B105] Rockson S. G. Kramer P. Razavi M. Szuba A. Filardo S. Fitzgerald P. (2000). Photoangioplasty for Human Peripheral Atherosclerosis. Circulation 102, 2322–2324. 10.1161/01.CIR.102.19.2322 11067782

[B106] Saba I. Jakubowska W. Bolduc S. Chabaud S. Dai Y. Zhao X. (2018). Engineering Tissues without the Use of a Synthetic Scaffold: A Twenty-Year History of the Self-Assembly Method. BioMed Res. Int. 2018, 1–13. 10.1155/2018/5684679 PMC5863296 29707571

[B107] Sadaksharam J. Nayaki K. P. T. Panneer Selvam N. (2012). Treatment of Oral Lichen Planus with Methylene Blue Mediated Photodynamic Therapy - a Clinical Study. Photodermatol. Photoimmunol. Photomed. 28, 97–101. 10.1111/j.1600-0781.2012.00647.x 22409713

[B109] Shao C. Liu Y. Chi J. Ye F. Zhao Y. (2021). Hierarchically Inverse Opal Porous Scaffolds from Droplet Microfluidics for Biomimetic 3D Cell Co-culture. Engineering 7, 1778–1785. 10.1016/j.eng.2020.06.031

[B110] Sharma R. Kamal A. Abdinejad M. Mahajan R. K. Kraatz H.-B. (2017). Advances in the Synthesis, Molecular Architectures and Potential Applications of Gemini Surfactants. Adv. Colloid Interface Sci. 248, 35–68. 10.1016/J.CIS.2017.07.032 28800974

[B111] Shin H. T. Kim J. H. Shim J. Lee J. H. Lee D. Y. Lee J. H. (2015). Photodynamic Therapy Using a New Formulation of 5-aminolevulinic Acid for Wrinkles in Asian Skin: A Randomized Controlled Split Face Study. J. Dermatological Treat. 26, 246–251. 10.3109/09546634.2014.933163 24913131

[B112] Song H.-H. G. Park K. M. Gerecht S. (2014). Hydrogels to Model 3D *In Vitro* Microenvironment of Tumor Vascularization. Adv. Drug Deliv. Rev. 79-80, 19–29. 10.1016/j.addr.2014.06.002 24969477 PMC4258430

[B113] Spring B. Q. Rizvi I. Xu N. Hasan T. Hospital M. G. Medical H. (2016). The Role of Photodynamic Therapy in Overcoming Cancer Drug Resistance. Photochem. Photobiol. Sci. 14, 1476–1491. 10.1039/c4pp00495g PMC4520758 25856800

[B114] Sternberg E. D. Dolphin D. Brückner C. (1998). Porphyrin-based Photosensitizers for Use in Photodynamic Therapy. Tetrahedron 54, 4151–4202. 10.1016/S0040-4020(98)00015-5

[B137] Stender I. M. Na R. Fogh H. Gluud C. Wulf H. C. (2000). Photodynamic Therapy With 5-Aminolaevulinic Acid or Placebo for Recalcitrant Foot and Hand Warts: Randomised Double-Blind Trial. Lancet (London, England) 355 (9208), 963–966. 10.1016/S0140-6736(00)90013-8 10768434

[B115] Sun X. Cao Z. Mao K. Wu C. Chen H. Wang J. (2020). Photodynamic Therapy Produces Enhanced Efficacy of Antitumor Immunotherapy by Simultaneously Inducing Intratumoral Release of Sorafenib. Biomaterials 240, 119845. 10.1016/j.biomaterials.2020.119845 32085974

[B116] Tarassoli S. P. Jessop Z. M. Al-Sabah A. Gao N. Whitaker S. Doak S. (2018). Skin Tissue Engineering Using 3D Bioprinting: An Evolving Research Field. J. Plastic, Reconstr. Aesthetic Surg. 71, 615–623. 10.1016/j.bjps.2017.12.006 29306639

[B117] Tawakol A. Castano A. P. Anatelli F. Bashian G. Stern J. Zahra T. (2006). Photosensitizer delivery to vulnerable atherosclerotic plaque: comparison of macrophage-targeted conjugate versus free chlorine(e6). J. Biomed. Opt. 11, 021008. 10.1117/1.2186039 16674183 PMC2936819

[B118] Tedesco A. C. Primo F. L. Jesusda P. d. C. C. d. C. C. (2017). “Antimicrobial Photodynamic Therapy (APDT) Action Based on Nanostructured Photosensitizers,” in Multifunctional Systems for Combined Delivery, Biosensing and Diagnostics (Amsterdam, Netherlands: Elsevier), 9–29. 10.1016/B978-0-323-52725-5.00002-2

[B119] Tedesco A. Jesus P. (2017). Low Level Energy Photodynamic Therapy for Skin Processes and Regeneration. Photomed. - Adv. Clin. Pract. 10.5772/65344

[B120] van Dijk E. H. C. Fauser S. Breukink M. B. Blanco-Garavito R. Groenewoud J. M. M. Keunen J. E. E. (2018). Half-Dose Photodynamic Therapy versus High-Density Subthreshold Micropulse Laser Treatment in Patients with Chronic Central Serous Chorioretinopathy. Ophthalmology 125, 1547–1555. 10.1016/j.ophtha.2018.04.021 29776672

[B121] Webber M. J. (2016). Engineering Responsive Supramolecular Biomaterials: Toward Smart Therapeutics. Bioeng. Transl. Med. 1 (3), 252–266. 10.1002/btm2.10031 29313016 PMC5689538

[B122] White M. D. Mallucci G. R. (2009). Therapy for Prion Diseases: Insights from the Use of RNA Interference. Prion 3, 121–128. 10.4161/pri.3.3.9289 19597349 PMC2802775

[B123] Woappi Y. Altomare D. Creek K. E. Pirisi L. (2020). Self-assembling 3D Spheroid Cultures of Human Neonatal Keratinocytes Have Enhanced Regenerative Properties. Stem Cell. Res. 49, 102048. 10.1016/J.SCR.2020.102048 33128954 PMC7805020

[B124] Wolter J. R. Meyer R. F. (1985). Sessile Macrophages Forming Clear Endotheliumlike Membrane on the inside of Successful Keratoprosthesis. Graefe's Arch. Clin. Exp. Ophthalmol. 222, 109–117. 10.1007/BF02173533 3979831

[B125] Wu R. W. K. Chu E. S. M. Yuen J. W. M. Huang Z. (2020a). Comparative Study of FosPeg Photodynamic Effect on Nasopharyngeal Carcinoma Cells in 2D and 3D Models. J. Photochem. Photobiol. B Biol. 210, 111987. 10.1016/j.jphotobiol.2020.111987 32801063

[B126] Yakavets I. Jenard S. Francois A. Maklygina Y. Loschenov V. Lassalle H.-P. (2019). Stroma-rich Co-culture Multicellular Tumor Spheroids as a Tool for Photoactive Drugs Screening. J. Clin. Med. 8, 1686. 10.3390/jcm8101686 PMC6832590 31618880

[B127] Yang G.-L. Zhao M. Wang J.-M. He C.-F. Luo Y. Liu H.-Y. (2013). Short-term Clinical Effects of Photodynamic Therapy with Topical 5-aminolevulinic Acid for Facial Acne Conglobata: An Open, Prospective, Parallel-Arm Trial. Photodermatol. Photoimmunol. Photomed. 29, 233–238. 10.1111/phpp.12059 24001378

[B128] Yoo S. W. Oh G. Ahn J. C. Chung E. (2021). Non-Oncologic Applications of Nanomedicine-Based Phototherapy. Biomedicines 9, 113. 10.3390/biomedicines9020113 33504015 PMC7911939

[B129] Zangirolami A. C. Dias L. D. Blanco K. C. Vinagreiro C. S. Inada N. M. Arnaut L. G. (2020). Avoiding Ventilator-Associated Pneumonia: Curcumin-Functionalized Endotracheal Tube and Photodynamic Action. Proc. Natl. Acad. Sci. U.S.A. 117, 22967–22973. 10.1073/pnas.2006759117 32868444 PMC7502737

[B130] Zhang J. Jiang C. Figueiró Longo J. P. Azevedo R. B. Zhang H. Muehlmann L. A. (2018). An Updated Overview on the Development of New Photosensitizers for Anticancer Photodynamic Therapy. Acta Pharm. Sin. B 8, 137–146. 10.1016/j.apsb.2017.09.003 29719775 PMC5925394

[B131] Zhang Z. Lu X. N. Liang J. Tang H. Yang Y. S. Zhu X. H. (2015). Evaluation of Photodynamic Therapy Using Topical Aminolevulinic Acid Hydrochloride in the Treatment of Condylomata Acuminate. Int. J. Clin. Exp. Med. 8, 6517–6521. 26131281 PMC4483838

[B133] Zhao Y. Tu P. Zhou G. Zhou Z. Lin X. Yang H. (2016). Hemoporfin Photodynamic Therapy for Port-Wine Stain: A Randomized Controlled Trial. PLoS One 11, e0156219. 10.1371/journal.pone.0156219 27227544 PMC4881994

[B135] Zhou Z. Zhang L. Zhang Z. Liu Z. (2021). Advances in Photosensitizer-Related Design for Photodynamic Therapy. Asian J. Pharm. Sci. 16, 668–686. 10.1016/j.ajps.2020.12.003 35027948 PMC8737425

[B136] Zhuang P. Sun A. X. An J. Chua C. K. Chew S. Y. (2018). 3D Neural Tissue Models: From Spheroids to Bioprinting. Biomaterials 154, 113–133. 10.1016/j.biomaterials.2017.10.002 29120815

